# Examining the influence of treatment preferences on attrition, adherence and outcomes: a protocol for a two-stage partially randomized trial

**DOI:** 10.1186/s12912-015-0108-4

**Published:** 2015-11-09

**Authors:** Souraya Sidani, Mary Fox, David L. Streiner, Joyal Miranda, Suzanne Fredericks, Dana R. Epstein

**Affiliations:** School of Nursing, Ryerson University, 350 Victoria Street, Toronto, ON M5B 2 K3 Canada; School of Nursing, York University, Toronto, ON Canada; Department of Psychiatry and Behavioural Neurosciences, McMaster University, Hamilton, ON Canada; Nursing Research and Evidence-Based Practice, Phoenix Veterans Affairs Health Care System, Phoenix, AZ USA

**Keywords:** Treatment preference, Two-stage randomized clinical trial, Behavioral therapy, Insomnia, Adherence to treatment, Attrition, Outcomes

## Abstract

**Background:**

Empirical evidence pertaining to the influence of treatment preferences on attrition, adherence and outcomes in intervention evaluation trials is inconsistent. The inconsistency can be explained by the method used for allocating treatment and measuring preferences. The current methodological study is designed to address these factors by implementing the two-stage partially randomized or preference trial design, and administering a validated measure to assess participants’ preferences for the treatments under evaluation. It aims to compare attrition, adherence and outcomes for participants allocated randomly or by preference to treatment. The study is in its final stages of data collection; its protocol is presented in this paper.

**Methods/Design:**

A partially randomized clinical or preference trial is used. Eligible participants are randomized to two trial arms. First is the random arm involving random assignment to treatments, and second is the preference arm involving allocation to the chosen treatment. Participants with chronic insomnia are targeted. Two behavioral treatments are offered, stimulus control therapy and sleep restriction therapy, in the same format (small group) and dose (two sessions given over a 4-week period). A participant log is used to collect data on attrition. Adherence is evaluated in terms of exposure and enactment of treatment. Sleep-related outcomes (sleep parameters and perceived insomnia severity) are measured at pretest, posttest, 6 and 12 month follow-up. Treatment preferences, adherence and outcomes are assessed with reliable and valid measures.

**Discussion:**

The advantages and limitations of the preference trial design are highlighted. The challenges in implementing the trial are discussed relative to the distribution of participants in the groups defined by treatment received and method of treatment allocation.

**Trial registration:**

ClinicalTrials.gov Registry NCT02513017

## Background

Treatment preferences represent people’s choice of therapy [1]. Treatment preferences contribute to attrition, adherence and outcomes of treatment [2] and they can jeopardize validity [3] in intervention research. The empirical evidence pertaining to the influence of preferences on attrition, adherence and outcomes is inconsistent. In addition to differences in treatments and target populations, two methodological factors could explain the inconsistency in findings: the method used to assign participants to treatment and the measure used to assess treatment preferences. The current study, which is in its final stage of collecting follow-up data, is designed to address the methodological factors. This is accomplished by (1) implementing the two-stage partially randomized or preference trial design, which involves two methods (random and preference-based) of assignment to treatment and hence, facilitates the examination of the influence of treatment preferences on attrition, adherence and outcomes [4] within and across treatment groups; and (2) administering a validated measure to assess participants’ preferences for the treatments under evaluation.

This paper describes the study protocol for conducting the two-stage partially randomized clinical or preference trial. The paper begins with an explanation of the rationale for the study. The trial protocol is described, and challenges in its implementation are presented. The advantages and limitations of the design are also discussed.

The influence of treatment preferences on attrition, adherence, and outcomes has been investigated in several studies evaluating medical, behavioral and educational interventions for a variety of clinical conditions. The studies’ findings were synthesized in systematic reviews and meta-analyses. Overall, they point to inconsistencies as summarized next.

Three individual studies [5–7] and one systematic review [8] reported comparable attrition rates for participants who received treatment that did and did not match their preference. However, the results of two studies [7, 9] and two meta-analyses [10, 11] showed lower attrition rates for the matched than the mismatched subgroups, implying that participants who received their preferred treatment were less likely to withdraw from the trial.

The results of five individual studies were consistent in showing that assignment to treatment, matched to participants’ preferences, is associated with enhanced adherence operationalized as attendance at treatment sessions [9, 12–14] or engagement in treatment activities [15]. In contrast, the findings of the two systematic reviews did not support the relationship between preferences and adherence [16, 17].

The findings of one systematic review [8] and two meta-analyses indicated that participants allocated to the preferred treatment demonstrated greater improvement in outcomes than those randomized to the non-preferred treatment. The mean effect size (Cohen’ d coefficient) ranged between .15 [95 % confidence interval: .01–.31] [10] and .31 [95 % confidence interval: .20–.43] [11]. In contrast, Gelhorn and colleagues [16] concluded that preferences had minimal impact on the outcomes.

Conceptual and methodological factors could have contributed to the inconsistent findings and the small-to-moderate effect sizes quantifying the impact of treatment preferences on outcomes. These factors include differences in the characteristics of the target populations (e.g. patients with depression or heart disease), the types of treatment under investigation (e.g. medical, behavioral), the context of treatment implementation (e.g. institution, home), the method of allocation to treatment, and the method for assessing preferences. The latter two methodological factors are of concern because they could have attenuated the estimates of the association between preferences and outcomes and they can be addressed in future research aimed to evaluate the influence of treatment preferences. Most previous studies consisted of randomized clinical trials in which participants, whose preferences were assessed at baseline, were randomized to treatment groups. The random method of assignment ignored participants’ preferences; however, these preferences were accounted for at the stage of data analysis by generating a match-mismatch (i.e. whether the received treatment was congruent with the preferred treatment) between-subject factor. The extent to which active involvement of participants in treatment selection and allocation to the preferred treatment make a difference in attrition, adherence, and outcomes has not been extensively investigated.

In the majority of trials reviewed, the method for assessing preferences consisted of administering one item inquiring about participants’ choice, which has three limitations. First, the use of one item, with no demonstrated psychometric properties, could have introduced measurement error, raising questions about the accuracy of the expressed treatment preference in reflecting participants’ choice. Second, the trials’ reports provided minimal details regarding the treatment-related information presented to participants prior to eliciting their preferences, and the format in which such information was given. The content and format for presenting treatment-related information affect participants’ perception of the treatment options [18–20].

Third, single items do not capture the complexity of treatment preferences, which are based on careful evaluation of the treatments’ attributes such as effectiveness, severity of side effects, and convenience of implementing the treatment in daily life [21–23]. Therefore, the method for assessing preferences has to be systematic in order to obtain well-informed and accurate expressions of participants’ preferences.

The two methodological factors were addressed in the current study. The method of allocation to treatment was examined by applying the two-stage partially randomized clinical trial [4] that involved two methods of allocation: random and preference-based. A systematic method, operationalized in the Treatment Acceptability and Preference scale [24], was followed to assess treatment preferences.

The goal of the current study was to clarify the contribution of treatment preferences in intervention evaluation research. The specific study objectives were:

1. To compare participants randomized to treatment and participants allocated to the preferred treatment on: attrition, adherence to treatment, and outcomes.
2. Among randomized participants, to compare those who received matched (i.e. congruent with expressed preferences) and those who received mismatched (i.e. incongruent with expressed preferences) treatment on: attrition, adherence to treatment, and outcomes.

## Methods

### Design

**Fig. 1:**
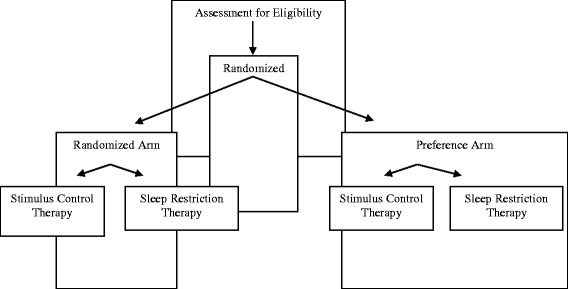
Illustration of the PRCT design

A two-stage partially randomized clinical or preference trial (PRCT), originally described by Rücker [4], was used. The trial protocol was approved by the Research Ethics Board at Ryerson University (Protocol Reference # 2010-085-3). The research performed on participants were in compliance with the Helsinki Declaration. As illustrated in Fig. 1, the PRCT involves two stages for participants’ allocation to treatment. In the first stage, eligible participants that have provided written informed consent are randomized to the random or preference arms of the trial, after obtaining pretest data. Randomization at this stage increases the likelihood that participants in the two arms are comparable in their socio-demographic and clinical profiles, status on pretest outcomes, and expressed preferences for the treatments under evaluation. In the second stage, the method of assignment to treatment differs between the trial arms. In the random arm, participants are randomly assigned to treatment, whereas in the preference arm, participants are allocated to the preferred treatment. This treatment allocation scheme allows comparisons on relevant variables between participants who receive the same treatment on the basis of either chance or preference; the results of these comparisons determine the contribution of preferences.

The treatments investigated in the current study are two behavioral therapies that demonstrated efficacy in the management of chronic insomnia: stimulus control therapy and sleep restriction therapy [25]. Eligible consenting participants complete the pretest questionnaire measuring socio-demographic and clinical characteristics, sleep outcomes, perception of the treatments’ attributes and preferences for the behavioral therapies. Participants are randomized to the two trial arms, using sealed opaque envelopes. Participants in the random arm are randomly assigned to the stimulus control therapy or the sleep restriction therapy, using sealed envelopes. Participants in the preference arm are allocated to the treatment they indicate that they prefer. Participants expressing no preferences are randomly assigned to treatment. The outcomes are assessed at four points in time: pretest (i.e. within 2 weeks before treatment); post-test (i.e. within 1 week after treatment completion); 6-month and 12-month follow-up. Data on adherence to treatment are collected prospectively, during the treatment and post-treatment periods. Three strategies are used to minimize attrition: reimbursement for travel expenses to attend the data collection and the treatment sessions, a $10 gift certificate, and telephone call reminders. The calls are made to all participants within 1 week prior to the planned research activity (i.e. attendance at treatment session and data collection).

### Sample

Participants with insomnia are recruited through community media advertisements in local newspapers, radio station, and local newsletters targeting middle-aged and older adults. Flyers advertising the study are distributed to community health centers, hospital outpatient clinics, a leisure complex for older adults and sleep clinics.

Persons with insomnia are eligible for the study if they 1) are non-institutionalized adults (≥40 years of age) as insomnia is prevalent in middle-aged and older adults [26], 2) have the ability to read and write English, and 3) complain of difficulty initiating and/or difficulty maintaining sleep, manifested in sleep onset latency and/or time awake after sleep onset of 30 min or more per night, for a minimum of three nights per week, for a duration of at least 6 weeks [27], ascertained with relevant questions of the Insomnia Interview Schedule [28]. Individuals are excluded if they have: 1) cognitive impairment indicated by a score < 24 on the Mini-Mental State Exam [29] because cognitive impairment interferes with participants’ understanding of and ability to implement the behavioral therapies; and 2) confirmed medical diagnosis of sleep apnea as reported by participants; the behavioral therapies are not recommended for sleep apnea.

The sample size is estimated to attain adequate power to determine the effects of treatment preferences on attrition, adherence, and sleep outcomes. Sample size estimation also considers the repeated measure feature of the design. Within-subject designs require a smaller number of participants than between-subject designs, to achieve the same statistical power [30]. A medium sized effect (0.5) is anticipated on the basis of: 1) results of a systematic review showing that behavioral therapies had moderate-to-large effects on sleep parameters (sleep onset latency and wake after sleep onset) in middle-aged and older adults [31]; and 2) an expected medium effect for the comparison of participants assigned to treatment randomly or congruent with their preferences. A medium effect is anticipated, rather than a small effect as reported in the literature, with the use of a reliable and valid measure of treatment preferences. Applying Cohen’s [32] criteria for an anticipated medium effect size for the main comparison, the inclusion of four groups (i.e. participants allocated to the stimulus control therapy and those allocated to the sleep restriction therapy either randomly or based on their preference), and setting α at .05 and β at .80, the required sample size is 60 per group, for a total of 240 participants to be recruited.

### Behavioral therapies for insomnia

Stimulus control therapy is a behavioral treatment for the management of chronic insomnia. It aims to assist persons with insomnia to re-associate the bed and the bedroom with falling asleep or back to sleep, and to acquire a consistent sleep pattern. This therapy entails specific instructions that focus on developing new sleep habits, such as avoiding activities (e.g. reading, watching TV, and worrying) in bed, getting out of bed if unable to fall asleep or back to sleep within 15–20 min and engaging in quiet activities until sleepy, going to bed only when sleepy, and waking up at the same time everyday.

Sleep restriction therapy consists of limiting the amount of time spent in bed to a specific sleep time. Sleep time is individualized, based on the persons’ total sleep time identified by reviewing the daily sleep diary kept at pretest. A sleep-wake schedule is planned to fit the persons’ lifestyle. The sleep-wake schedule is changed to accommodate improvements in the persons’ sleep, over time.

Both treatments are given by Master’s prepared therapists, trained in the implementation of the behavioral therapies. The sessions are offered in a small group (4–6 persons) format, based on availability of participants and to avoid any delay in treatment receipt. The treatments are delivered in the same dose: two sessions of 90 min each, once every 15 days, over a 4-week treatment period.

The results of two systematic reviews consistently demonstrated the effectiveness of stimulus control therapy and sleep restriction therapy [25, 31]. The treatments’ effects were reported in the short (1–3 months), intermediate (6 months), and long (≥12 months) term follow-up [25].

### Variables and measures

*Socio-demographic characteristics* of participants are assessed with standard questions gathering information on age, sex, marital status, level of education, employment status, and ethnicity.

*Clinical characteristics* relate to the type of insomnia (i.e. difficulty falling or staying asleep), whether the insomnia is primary (not associated with the onset of physical or mental health problems) or comorbid (associated with the occurrence of such problems), and duration of insomnia. These are assessed with relevant items of the Insomnia Interview Schedule developed and validated by Morin [28]. Participants also report on the presence of comorbid conditions (e.g. cancer, diabetes, cardiac disease) and on the type of treatment for managing these conditions, which could affect the experience of insomnia and the expressed preferences for insomnia treatment, and the effectiveness of treatment in managing insomnia.

*Preferences for treatment* are measured with an adapted version of the Treatment Acceptability and Preference scale [24]. It contains three sections. The first presents the description of one treatment. The description introduces the treatment’s name and goal; explains the activities comprising the treatment, the recommendations to be followed by the participants, the mode of delivery and dose of treatment; and summarizes evidence of the treatment’s benefits and risks. Information on benefits and risks is synthesized from available empirical literature, and presented in simple, non-technical, and easily understood terms. Following the description is a set of items for participants to rate the treatment relative to four attributes: 1) effectiveness in managing insomnia in the short and long term, and in improving daytime functioning; 2) appropriateness or suitability in addressing insomnia; 3) severity of side effects; and 4) convenience, that is, ease of implementing it in daily life. A five-point rating scale, ranging from *not at all* (0) to *very much* (4) is used. The second section of the Treatment Acceptability and Preference (TAP) presents the description of the alternative treatment, and the items for rating it. The third section includes two questions asking participants to indicate if they have a preference for any treatment and, if they do, the preferred treatment. In this study, the nine items rating the attributes of the two behavioral therapies are internally consistent (Cronbach’s alphas > .85) and valid, evidenced by significant associations between the treatments’ ratings and the expressed preferences. The order for presenting the treatment options is randomized to avoid an order effect.

*Attrition* is noted and documented in a log. The research assistants record the following information for each participant: completion of pretest measures, attendance at treatment sessions, completion of post-test, 6-month and 12-month follow-up measures, time at which the participant withdraw and reasons for withdrawal as reported by participants. Attrition rate will be computed as the percentage of participants who drop out of the study at any time after providing consent to take part in the trial.

*Two components of adherence to treatment* are examined: exposure and enactment. Exposure is operationalized as participants’ attendance at the planned treatment sessions. The therapist facilitating the sessions records the presence of each participant and the total number of sessions attended will be counted. Enactment represents the extent to which participants apply the treatment recommendations in daily life. It is assessed by having participants complete a checklist. The checklist was developed following the procedure described by Stein and colleagues [33]. It includes 13 items reflecting the behavioral treatments’ recommendations that are discussed during the sessions and that participants are expected to carry out on a daily basis. Examples of these recommendations include: avoiding caffeine and nicotine before bedtime, going to bed only when sleepy and waking up at a regular time. Participants indicate whether or not they implement each of the listed treatment recommendations on every day of the treatment and post-test periods. They return the completed checklist along with the daily sleep diary every morning. The percentage of treatment recommendations actually applied, averaged by week, will quantify enactment.

*Sleep outcomes* include sleep parameters and perceived insomnia severity. The sleep parameters are assessed with the daily sleep diary (DSD) completed for a week, at each data collection point and during the 4-week treatment period. The DSD is a self-administered log of nightly sleep behaviors, developed by Morin [28]. Participants report on bed time, time to fall asleep, number and length of each awakening, and wake up time. They complete the DSD upon awakening and are asked to return the completed DSD by either calling in their responses to a voice mail service or sending them by e-mail, each morning to decrease the possibility of retrospective estimates and recall bias. The DSD demonstrated test-retest reliability (r: .69–.93) and validity, evidenced by correlation with actigraphy values for the same variables [27]. Relevant DSD data will be used to quantify the following sleep parameters: sleep onset latency (i.e. time it takes to fall asleep), wake after sleep onset (i.e. time awake across all awakenings), total sleep time, and sleep efficiency (i.e. percentage of total time in bed actually asleep).

Perceived insomnia severity is measured with the Insomnia Severity Index (ISI). The ISI has seven items assessing distress with the sleep problem and satisfaction with sleep patterns. The ISI demonstrated acceptable internal consistency reliability (α ≥ .85) and validity as evidenced by correlation with other subjective and objective measures of insomnia severity [34].

### Data analysis

In addition to descriptive statistics to characterize the participants in terms of their demographic and clinical characteristics, the following analyses are planned to address the study objectives:

- Objective 1: Chi-square test will be used to compare the number of participants who withdrew from the study across the four study groups defined by the type of treatment received (stimulus control therapy and sleep restriction therapy) and the method of allocation to treatment (random and preference). One-way analysis of variance will be applied to examine differences in level of adherence (i.e. attendance to treatment sessions and enactment of treatment recommendations), and the pattern of change (estimated with a slope) in the sleep outcomes, across groups.
- Objective 2: Chi-square test will compare the number of dropouts between participants in the random arm who receive matched and mismatched treatment. Independent samples t-test will examine differences between the matched and mismatched subgroups in levels of adherence (exposure and enactment) to treatment and the pattern of change in the sleep outcomes.

## Discussion

The study is in its final stage of collecting outcome data for the 12-month follow-up. To date, 421 persons showed interest and agreed to undergo initial screening.

The study extends previous research on the role of treatment preferences in intervention evaluation research in two ways. First, the two-stage PRCT design is applied, which is considered the most appropriate design for evaluating the influence of treatment preferences because of its advantages. The PRCT design is useful to dismantle the influence of treatment preferences from the impact of treatment on attrition, adherence and outcomes. This is accomplished by creating groups of participants who receive the same treatment randomly or by preference; comparison of these groups indicates the contribution of treatment preferences to attrition, adherence and outcomes. The PRCT design also minimizes the potential for selection bias resulting from non-comparability of participants assigned to different treatments randomly or on the basis of preference [3, 10]. The advantages stem from the randomization of participants to the random and preference arms in the first stage of assignment. Randomization at this stage maintains a balanced number and comparability on baseline characteristics of participants allocated to the two arms of the trial. This initial comparability reduces the potential for selection bias and confounding of the effects of treatments and preferences. It also enhances the validity of conclusions regarding the effectiveness of the treatments and the influence of preferences on adherence and outcomes of the treatments under investigation.

Second, the study uses a systematic method for assessing treatment preferences. The method begins by providing information on the treatments’ goals, components, activities, mode and dose of delivery, benefits and risks; it proceeds by having participants rate each treatment relative to four attributes prior to indicating their preferences. The TAP measure operationalizes this systematic method, has sound psychometric properties, and is expected to accurately capture participants’ preferences.

Despite its advantages, there are some challenges in carrying out a two-stage PRCT. The first challenge relates to the first stage of randomization. Through the process of obtaining informed consent, participants are informed of the two stages and of the method for treatment allocation within each arm. Participants with strong treatment preferences may be unwilling to leave treatment choice to chance and they may desire allocation to the preference arm. If randomized to the random arm, they may react unfavorably [4] and withdraw from the study.

The second challenge in the implementation of the two-stage PRCT relates to the method of treatment allocation applied in the second stage of the trial. At this stage, participants in the random arm are randomized to treatment, yielding a balanced number of participants assigned to the treatment groups. However, the equality of the treatment groups’ sizes is not achieved in the preference arm if most participants choose one treatment over the other. Large differences in group sizes may yield unequal within-group variances; if not addressed with appropriate statistical formula, unequal variances may reduce the power to detect significant treatment and preference effects [35]. The validity of these conclusions is further jeopardized if the pretest comparability of participants is not maintained. Randomization to treatment, applied in the random arm, increases the likelihood of achieving baseline comparability. However, such comparability may not be observed in the preference arm due to possible differences in the profile of participants choosing the alternative treatments under evaluation. Therefore, the baseline comparability of participants assigned to the different treatments randomly or by preference is not maintained, potentially confounding the results.

The third challenge in conducting the two-stage PRCT relates to the reactions of participants assigned to treatment on the basis of chance or preference. Specifically, participants in the random arm may have preferences for the treatments under investigation. Those randomized to the non-preferred treatment may react unfavorably. In contrast, participants in the preference arm may react favorably because they get the treatment they desire. These reactions may influence the participants’ adherence and responses to the allocated treatment.

Empirical evidence suggests that preferences for treatment contribute, to some extent, to participants’ attrition and adherence, and achievement of outcomes. Research designs, methods, and strategies are needed to account for treatment preferences and explore their influence on attrition, adherence, and outcome achievement with the ultimate goal of enhancing validity of conclusions in intervention evaluation research. The two-stage PRCT design is a viable alternative that allows for comparisons of participants representing the same target population and assigned to the same treatment on the basis of chance or preference. Just like other designs, the two-stage PRCT has advantages and limitations, and its application poses some challenges. The challenges relate to ignoring participants’ preferences in the random arm of the trial; this in turn, may trigger a sense of disappointment and dissatisfaction associated with depriving participants with the act of choosing the treatment.

Assignment to the preferred treatment within the context of intervention research is consistent with the process of treatment selection advocated and often applied in the context of day-to-day practice. In the latter context in which the patient-centered approach to care is emphasized, patients are informed of alternative treatments for the management of the presenting clinical problem, involved in treatment decision making, and provided the treatment that is responsive to their needs and preferences [36]. Results of intervention research that account for participants’ treatment preferences point to the consequences of providing treatments that are responsive to their desire and thus, are relevant to practice.
